# D-Lactate altered mitochondrial energy production in rat brain and heart but not liver

**DOI:** 10.1186/1743-7075-9-6

**Published:** 2012-02-01

**Authors:** Binbing Ling, Fei Peng, Jane Alcorn, Katharina Lohmann, Brian Bandy, Gordon A Zello

**Affiliations:** 1 College of Pharmacy and Nutrition, University of Saskatchewan, Saskatoon, SK, Canada; 2 Large Animal Clinical Sciences, Western College of Veterinary Medicine, Saskatoon, SK, Canada

**Keywords:** D-Lactate, Mitochondrial function, Rat, Brain, Heart

## Abstract

**Background:**

Substantially elevated blood D-lactate (DLA) concentrations are associated with neurocardiac toxicity in humans and animals. The neurological symptoms are similar to inherited or acquired abnormalities of pyruvate metabolism. We hypothesized that DLA interferes with mitochondrial utilization of L-lactate and pyruvate in brain and heart.

**Methods:**

Respiration rates in rat brain, heart and liver mitochondria were measured using DLA, LLA and pyruvate independently and in combination.

**Results:**

In brain mitochondria, state 3 respiration was 53% and 75% lower with DLA as substrate when compared with LLA and pyruvate, respectively (p < 0.05). Similarly in heart mitochondria, state 3 respiration was 39% and 86% lower with DLA as substrate when compared with LLA or pyruvate, respectively (p < 0.05). However, state 3 respiration rates were similar between DLA, LLA and pyruvate in liver mitochondria. Combined incubation of DLA with LLA or pyruvate markedly impaired state 3 respiration rates in brain and heart mitochondria (p < 0.05) but not in liver mitochondria. DLA dehydrogenase activities were 61% and 51% lower in brain and heart mitochondria compared to liver, respectively, whereas LLA dehydrogenase activities were similar across all three tissues. An LDH inhibitor blocked state 3 respiration with LLA as substrate in all three tissues. A monocarboxylate transporter inhibitor blocked respiration with all three substrates.

**Conclusions:**

DLA was a poor respiratory substrate in brain and heart mitochondria and inhibited LLA and pyruvate usage in these tissues. Further studies are warranted to evaluate whether these findings support, in part, the possible neurological and cardiac toxicity caused by high DLA levels.

## Introduction

Lactate exists as two stereoisomers, L-lactate and D-lactate. Under healthy physiological conditions, L-lactate is the major enantiomer found in blood whereas D-lactate is normally present in very low concentrations [1]. However, supra-physiological levels of D-lactate have been found in several disease states such as diarrhea, short bowel syndrome, and diabetes [2,3]. Most research in this area focus on the cause and the consequences of extremely high levels of D-lactate (> 3 mM D-lactate in plasma, resulting in D-lactic acidosis) in the body [3-7]. Although sub-clinical levels of D-lactate (high D-lactate levels, no acidosis) have been reported in several chronic diseases including diabetes and chronic fatigue syndrome [8,9], few studies explore the potential negative outcomes of such sub-clinical concentrations of D-lactate circulation in the body [10]. Interestingly, the clinical symptoms due to high levels of D-lactate (D-lactic acidosis) are similar to inherited or acquired abnormalities of pyruvate metabolism [11]. Therefore, D-lactate may directly or indirectly interfere pyruvate metabolism pathways, which are essential for mitochondrial energy production [12]. Any disturbance in pyruvate metabolism pathways may eventually impair mitochondrial energy generation and thus affect organs that are more highly energy dependent [13,14].

The brain and heart are metabolically active organs with substantial energy requirements. The major cellular pathways of energy production are glycolysis and mitochondrial oxidative phosphorylation [15]. During glycolysis, glucose is converted to pyruvate, which is accompanied by the production of ATP and NADH [15]. In mammalian cells, the enzymes responsible for pyruvate metabolism are located in the mitochondria [16]. Thus, pyruvate generated during glycolysis is transported into the mitochondria via monocarboxylate transporters (MCTs) particularly MCT1 [17]. In the mitochondria, pyruvate breakdown irreversibly funnels the products of glycolysis into the Krebs cycle to produce ATP and a large quantity of NADH [12]. NADH produced by both processes is then used to fuel mitochondrial ATP synthesis via oxidative phosphorylation or mitochondrial respiratory chain phosphorylation [15,16].

In some tissues, L-lactate oxidation can provide cellular energy in addition to glycolysis [18,19]. For example, L-lactate has been identified as the preferential oxidative energy substrate for the brain during excitation [20]. The Astrocyte-Neuron Lactate Shuttle hypothesis suggests that fuel for increased energy requirement of neurons during excitation is supplied by L-lactate from the surrounding astrocytes rather than glucose [18,21]. Following its transport into the cell, cytosolic L-lactate is converted to pyruvate by L-lactate dehydrogenase (LDH), an enzyme using NAD as a cofactor [18], which subsequently enters the TCA cycle in the mitochondria for further energy production. In addition, mitochondria also contain significant amounts of LDH, located largely in the inter-membrane space [18]. L-lactate transported into the mitochondria via MCTs is metabolized to pyruvate for energy production. Therefore, mitochondrial utilization of both L-lactate and pyruvate is crucial for cellular bioenergetics.

D-Lactate, recognized by MCTs [22], can competitively inhibit L-lactate and/or pyruvate transport via MCTs at the cellular level. For example, D-lactate inhibited L-lactate uptake into erythrocytes and brain cells and pyruvate uptake into cardiac myocytes [23,24]. Once D-lactate has entered the cells, it can affect the transport of L-lactate and/or pyruvate into the mitochondria and thus affects the usage of pyruvate and/or lactate. In fact, D-lactate interference of pyruvate metabolism has been postulated based on the clinical similarities between D-lactic acidosis and inherited or acquired abnormalities of pyruvate metabolism [25]; however, no experimental data are available to support this inference.

Cellular D-lactate is metabolized by mitochondrial D-lactate dehydrogenase (DDH) using FAD as cofactor [26,27]. The expression of DDH is tissue dependent and therefore affects the usage of D-lactate in different tissues [26,27]. For example, due to low expression levels of DDH in the brain, the rate of oxidation of D-lactate in the brain is considerably slower compared to that of L-lactate [26,27]. D-Lactate accumulation, then, may compromise energy metabolism by interfering with the mitochondrial usage of its more efficient energy substrates pyruvate and/or L-lactate and thus lead to toxicity. Energy deficiency in the brain of chickens has been reported following intracerebral infusion of D-lactate which suggests D-lactate's involvement in altered substrate utilization and ATP generation [28]. In this study, we hypothesized that D-lactate interferes with mitochondrial utilization of L-lactate and pyruvate.

The objectives of this study were to compare the mitochondrial utilization of D-lactate to L-lactate and pyruvate in mitochondria from rat heart, liver and brain tissues. We also investigated the effects of D-lactate on mitochondrial respiration with L-lactate or pyruvate as substrates. The D- and L-lactate dehydrogenase activities from different tissue mitochondria were also measured. To test the role of LDH on the mitochondrial respiration of L-lactate, a LDH inhibitor, oxamate (OX) was used in rat liver and brain. Our investigations also employed an MCT inhibitor, α-cyano-4-hydroxycinnamate (CINN), to identify the role of mitochondrial MCTs in D-lactate, L-lactate and pyruvate metabolism in rat liver and brain.

## Materials and methods

### Animals and Chemicals

Male Wistar rats were obtained from Charles River Canada (St. Constant, PQ) and were housed in a temperature and humidity controlled facility (22°C ± 2°C) on a 12-hour light: dark cycle (0700 h - 1900 h). Rats had free access to food and water and were allowed a 7-day acclimatization period. Rats were provided a nutritionally adequate rat diet (Prolab^® ^RMH 3000, Purina, Inc., Richmond, IN) *ad libitum*. This work was approved by the University of Saskatchewan's Animal Research Ethics Board, and adhered to the Canadian Council on Animal Care guidelines for humane animal use. Chemicals, unless specified, were purchased from Sigma-Aldrich.

### Mitochondrial Isolation

Mitochondria were isolated from rat (250-300 g body weight, n = 6) brain, heart and liver following humane euthanasia (isoflurane anaesthesia and exanguination) and rapid removal of organs. Briefly, freshly isolated organs were homogenized using a glass-teflon homogenizer (brain and liver) or a Polytron homogenizer (heart) with an isolation medium containing 250 mM sucrose, 10 mM HEPES and 1 mM EGTA (pH 7.2). In the case of brain, the mitochondria were isolated in the presence of 0.1% fat-free bovine serum albumin (BSA). The homogenate was then centrifuged at 1000 g for 8 min at 4°C. The supernatant was collected and centrifuged at 10000 g for 10 min at 4°C. The pellet was collected and washed twice with washing medium containing 250 mM sucrose, 10 mM HEPES and 0.1 mM EGTA. The final pellets of mitochondria were suspended in 2 mL of isolation medium without EGTA [29-32].

### Oxygen Uptake Studies

Mitochondrial protein was measured as described by the biuret method using bovine serum albumin as standard [33]. Respiration rates of isolated mitochondria were measured with a Clark-type electrode (DW1; Hansatech Instruments Ltd, Norfolk, England) in a water-jacketed glass chamber with magnetic stirring. An oxygen electrode was used and the respiration chamber was kept constant at 30°C. Oxygen uptake measurements were carried out in 1 mL of medium containing 210 mM mannitol, 70 mM sucrose, 0.1 mM EDTA, 20 mM Tris/HCl, 3 mM MgCl_2_, 5 mM KH_2_PO_4_/K_2_HPO_4 _and 0.2% BSA (pH 7.4) [34]. Assays were performed in duplicate using fresh mitochondria. The respiratory parameters of the mitochondria were tested using a respiratory cocktail (containing 62.5 μM each of malate, glutamate, alpha-ketoglutarate and pyruvate), pyruvate + malate, L-lactate + malate, or D-lactate + malate. Pyruvate (10 mM) and D/L-lactate (5 mM) were added alone or in combination. To inhibit the mitochondrial monocarboxylate transporter (mMCT), 5 mM α-cyano-4-hydroxycinnamate (CINN) was used. To inhibit mitochondrial LDH, 50 mM oxamate (OX) was used. Respiration was initiated by the addition of 1 mg protein of the mitochondrial suspension to the reaction medium, and a conventional respiratory experiment with transitions from state 4 to 3 was performed.

Mitochondrial respiratory state 4 is the resting state which is differentiated by relatively slow oxygen uptake and no availability of ADP [35]. On the other hand, mitochondrial respiratory state 3 is the active state with high rates of oxygen uptake and sufficient ADP supply [35]. Thus, state 3 was initiated by adding ADP (final concentration 0.1 mM). Mitochondrial function was assessed by the respiratory control ratio (RCR) and ADP:O ratios [36]. These two parameters are well accepted as indicators of electron transport chain coupling to ATP synthesis and efficiency of oxidative phosphorylation in the presence of different substrates. The RCR values were calculated as the ratio of the respiratory rate in state 3, after addition of ADP, to the rate of oxygen uptake without ADP (state 4). The ratio between the amount of ADP phosphorylated and oxygen consumed (ADP/O ratio) was also calculated [37].

### L- and D-Lactate Dehydrogenase (LDH and DDH) Assay

The LDH and DDH assays were performed photometrically by means of a 96 well spectrophotometer at 600 nm [38] at 25°C. Briefly, the mitochondrial sample was incubated for 2 min in 2 mL of standard medium consisting of 0.2 mM sucrose, 10 mM KCl, 20 mM Hepes/Tris, pH 7.2, 1 mM MgCl_2 _in the presence of 30 μM phenazine methosulphate (PMS) and 50 μM dichloroindophenol (DCIP). DDH activity was determined by measuring the decrease in absorbance at 600 nm (A_600_) due to DCIP reduction when 15 mM D-lactate was added. The activity was expressed as nmol of DCIP reduced per min per mg of protein.

### Statistics

Results are presented as mean ± SEM. Respiration rates and RCR values were compared using one way ANOVA with Fisher's least significant difference (LSD) post hoc tests. The statistical differences between the values with the presence of different substrates and the values without addition of any of the test substrates are reported in tables (Table 1, 2 and 3). Due to the complexity, the rest of the statistical results are described in the results section rather than in the tables.

**Table 1:** Respiratory parameters^a ^of isolated rat liver mitochondria with pyruvate, D-lactate, or L-lactate or as combination.

Substrate^b^	State 4	State 3	RCR	ADP/O
No substrate	3.24 ± 0.28	3.37 ± 0.34	1.04 ± 0.02	
Cocktail^c^	4.11 ± 0.13	30.12 ± 2.02*	7.33 ± 0.46*	2.87 ± 0.09
Pyruvate	5.01 ± 0.56	19.17 ± 2.56*	3.78 ± 0.11*	2.56 ± 0.04
D-lactate	3.82 ± 0.24	14.11 ± 1.13*	3.69 ± 0.11*	2.47 ± 0.11
L-lactate	4.22 ± 0.19	15.02 ± 0.98*	3.56 ± 0.15*	2.30 ± 0.24
Pyruvate + LLA	6.20 ± 0.41	21.40 ± 1.59*	3.45 ± 0.06*	-
Pyruvate + DLA	3.86 ± 0.23	17.58 ± 2.21*	4.56 ± 0.51*	-
LLA + DLA	3.76 ± 0.21	11.78 ± 0.41	3.14 ± 0.07*	-

^a^Respiratory rates in nmol O_2_/mg mitochondrial protein per min.

^b^Substrate concentrations: pyruvate (10 mM + 2.5 mM malate), DLA or LLA (5 mM + 2.5 mM malate), and LLA+DLA (2.5 mM LLA and 2.5 mM DLA+ 2.5 mM malate).

^c^Cocktail contains 62.5 μM each of malate, glutamate, alpha-ketoglutarate and pyruvate.

State 4: The respiration state without ADP.

State 3: The respiration state with the addition of ADP.

RCR, Respiratory Control Ratio (state 3 rate:state 4 rate).

ADP/O ratio: The ratio between the amount of ADP phosphorylated and oxygen consumed.

*Significantly different from the values without substrate. Values were compared using one way ANOVA with LSD as post hoc test, α < 0.05.

**Table 2:** Respiratory parameters^a ^of isolated rat brain mitochondria with pyruvate, D-lactate, or L-lactate or as combination.

Substrate^b^	State 4	State 3	RCR	ADP/O
No substrate	3.56 ± 0.31	5.21 ± 0.14	1.49 ± 0.11	
Cocktail^c^	4.51 ± 0.49	29.43 ± 2.92*	6.61 ± 0.73*	2.92 ± 0.01
Pyruvate	3.59 ± 0.21	30.98 ± 2.42*	8.46 ± 0.55*	2.12 ± 0.15
D-lactate	3.18 ± 0.16	7.64 ± 0.56	2.39 ± 0.10	-
L-lactate	3.18 ± 0.16	16.13 ± 0.49*	4.66 ± 0.17*	1.88 ± 0.10
Pyruvate + LLA	3.16 ± 0.45	22.09 ± 4.42*	7.16 ± 1.57*	-
Pyruvate + DLA	4.07 ± 0.51	16.41 ± 2.85	3.94 ± 0.40	-
LLA + DLA	3.40 ± 0.34	5.41 ± 0.48	1.60 ± 0.08	-

^a^Respiratory rates in nmol O_2_/mg mitochondrial protein per min.

^b^Substrate concentrations: pyruvate (10 mM + 2.5 mM malate), DLA or LLA (5 mM + 2.5 mM malate), and LLA+DLA (2.5 mM LLA and 2.5 mM DLA+ 2.5 mM malate).

^c^Cocktail contains 62.5 μeach of malate, glutamate, alpha-ketoglutarate and pyruvate.

State 4: The respiration state without ADP.

State 3: The respiration state with the addition of ADP.

RCR: Respiratory Control Ratio (state 3 rate:state 4 rate).

ADP/O ratio: The ratio between the amount of ADP phosphorylated and oxygen consumed.

*Significantly different from the values without substrate. Values were compared using one way ANOVA with LSD as post hoc test, α < 0.05.

**Table 3:** Respiratory parameters^a ^of isolated rat heart mitochondria with pyruvate, D-lactate, L-lactate alone or as combination as substrates.

Substrate^b^	State 4	State 3	RCR	ADP/O
No substrate	3.07 ± 0.15	7.72 ± 0.55	2.52 ± 0.08	
Cocktail^c^	11.35 ± 1.52*	70.89 ± 5.89*	6.39 ± 0.59*	2.76 ± 0.02
Pyruvate	12.51 ± 1.19*	74.74 ± 6.22*	5.95 ± 0.22*	3.01 ± 0.02
D-lactate	4.62 ± 0.19	9.27 ± 1.31	2.01 ± 0.27	-
L-lactate	4.58 ± 0.58	15.17 ± 2.67	3.24 ± 0.21	2.11 ± 0.34
Pyruvate + LLA	4.30 ± 0.25	18.23 ± 1.52	4.23 ± 0.15*	-
Pyruvate + DLA	5.28 ± 0.42	15.03 ± 0.74	2.91 ± 0.21	-
LLA + DLA	3.57 ± 0.22	3.95 ± 0.26	1.11 ± 0.08	-

^a^Respiratory rates in nmol O_2_/mg mitochondrial protein per min.

^b^Substrate concentrations: pyruvate (10 mM + 2.5 mM malate), DLA or LLA (5 mM + 2.5 mM malate), and LLA+DLA (2.5 mM LLA and 2.5 mM DLA+ 2.5 mM malate).

^c^Cocktail contains 62.5 μeach of malate, glutamate, alpha-ketoglutarate and pyruvate.

State 4: The respiration state without ADP.

State 3: The respiration state with the addition of ADP.

RCR: Respiratory Control Ratio (state 3 rate:state 4 rate).

ADP/O ratio: The ratio between the amount of ADP phosphorylated and oxygen consumed.

* Significantly different from the values without substrate. Values were compared using one way ANOVA with LSD as post hoc test, α < 0.05.

## Results

### Pyruvate, D-lactate or L-lactate as substrate for mitochondrial respiration

To demonstrate the ability of rat mitochondria from brain, liver and heart to use pyruvate, L-lactate or D-lactate as substrates, oxygen uptake was measured under respiratory state 4 conditions and state 3 conditions in the presence of 0.1 mM ADP. Mitochondria isolated from brain, heart and liver readily oxidized pyruvate and L-lactate with a respiration control ratios (RCR) values higher than 5 with a respiratory substrate cocktail and ATP/O values higher than 2, which are the generally accepted as normal for carefully prepared mitochondria from liver, heart and brain (Tables 1, 2 and 3). However, mitochondria isolated from brain and heart did not oxidize D-lactate efficiently as demonstrated by low state 3 respiration rates and RCR values close to values recorded without substrate, as well as unmeasurable ADP/O ratios (p > 0.05, Table 2 and 3). Liver mitochondria oxidized D-lactate with a RCR of 3.67 and ADP/O ratio of 2.47 (Table 2), which is similar to the L-lactate and pyruvate (p > 0.05). When compared to brain and liver, mitochondria from heart had twice as high or greater state 3 and state 4 respiration when using a cocktail or pyruvate (p < 0.05), but not L-lactate. Pyruvate showed the highest RCR in brain mitochondria with the lowest in liver (brain > heart > liver, p < 0.05). The RCR values for L-lactate in brain mitochondria were significantly higher than that of the liver and heart mitochondria (p < 0.05).

### Effect of D-lactate or L-lactate on mitochondrial oxidation of pyruvate in liver, heart and brain

D-Lactate significantly impaired oxygen consumption caused by pyruvate in rat brain and heart mitochondria. The oxygen consumption rate inhibition caused by D-lactate was slightly stronger compared to L-lactate (e.g. 47% (p < 0.05) vs. 29% (p > 0.05) reduction in state 3 in the brain, respectively compared to pyruvate alone as substrate). Interestingly, co-administration of D-lactate or L-lactate did not change mitochondrial respiration using pyruvate as substrate in liver mitochondria (p > 0.05). In addition, D-lactate significantly decreased oxygen consumption caused by L-lactate in rat brain (67% reduction in state 3, p < 0.05) and heart (74% reduction in state 3, p < 0.05) mitochondria with a slight but non-significant change in liver (22% reduction in state 3, p > 0.05) mitochondria.

### DDH and LDH activities in brain, heart and liver mitochondria

**Figure 1:**
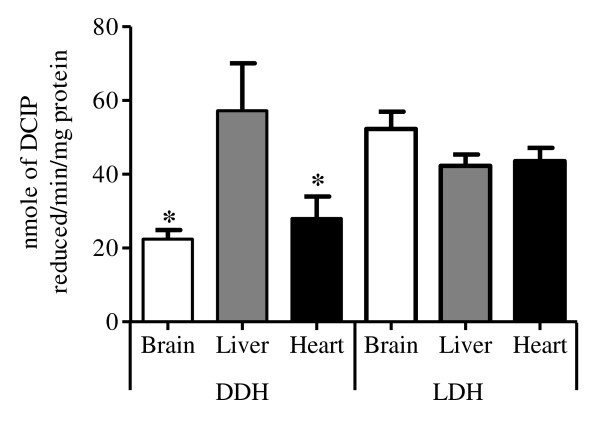
**The enzyme activities of DDH and LDH expressed as nmole of dichloroindophenol (DCIP) reduced/min/mg protein (Mean ± SEM) in mitochondria from rat brain, liver and heart (n = 6)**. * Different from liver mitochondria, P < 0.05.

DDH activity was significantly higher in rat liver mitochondria compared to rat brain and heart mitochondria (Figure 1). DDH activity in rat liver mitochondria was similar to LDH activity. The activity of LDH was similar in the brain, heart and liver mitochondria.

### Effect of oxamate (OX) and α-cyano-4-hydroxycinnamate (CINN) on mitochondrial respiration using pyruvate, D-lactate and L-lactate

In the presence of OX, a known LDH inhibitor, the oxygen consumption rate in state 3 and the RCR in brain and liver mitochondria were similar to the values without substrates (Table 4). In other words, mitochondrial L-lactate oxidation was completely blocked by OX. It is important to note that OX also partially inhibited respiration with pyruvate (p < 0.05) and D-lactate (p > 0.05) (e.g. 42% and 37% decrease respectively in state 3 in the brain) as substrate, which suggests that OX had other effects than just inhibition of LDH. In the presence of CINN, a known MCT inhibitor, the respirations caused by pyruvate, L-lactate and D-lactate were completely blocked in brain mitochondria with the state 3, state 4 respiration rate and RCR similar to values in the absence of substrate (Table 5).

**Table 4:** Respiratory parameters^a ^of isolated rat brain and liver mitochondria with pyruvate, D-lactate, L-lactate as substrates in the presence of oxamate, a LDH inhibitor.

Substrate^b^	Tissue	State 4	State 3	RCI
Pyruvate + OX	Brain	3.20 ± 0.57	17.96 ± 2.51	5.74 ± 0.41
	Liver	2.61 ± 0.09	9.98 ± 0.31	3.83 ± 0.05
LLA + OX	Brain	3.45 ± 0.12	4.27 ± 0.35	1.23 ± 0.06
	Liver	3.28 ± 0.23	4.19 ± 1.08	1.25 ± 0.23
DLA + OX	Brain	3.25 ± 0.15	4.83 ± 1.11	1.47 ± 0.30
	Liver	4.11 ± 0.15	14.34 ± 0.21	3.50 ± 0.12
Pyr +LLA+ OX	Brain	3.06 ± 0.42	7.14 ± 1.23	2.32 ± 0.14
	Liver	2.16 ± 0.22	2.73 ± 0.45	1.29 ± 0.25

^a^Respiratory rates in nmol O_2_/mg mitochondrial protein per min.

^b^Substrate concentrations: pyruvate (10 mM + 2.5 mM malate) and LLA or DLA (5 mM + 2.5 mM malate); OX, oxamate (50 mM).

State 4: The respiration state without ADP.

State 3: The respiration state with the addition of ADP.

RCR: Respiratory Control Ratio (state 3 rate:state 4 rate).

ADP/O ratio: The ratio between the amount of ADP phosphorylated and oxygen consumed.

*Significantly different from the values without substrate. Values were compared using one way ANOVA with LSD as post hoc test. α < 0.05

Note: Refer to Table 1 and 2 for the respiratory parameters without substrates or with cocktail.

**Table 5:** Respiratory parameters^a ^of isolated rat brain and liver mitochondria with pyruvate, D-lactate, L-lactate as substrates in the presence of CINN, a monocarboxylate transporter inhibitor.

Substrate^b^	Tissue	State 4	State 3	RCI
Pyruvate + CINN	Brain	3.42 ± 0.33	5.88 ± 0.72	1.72 ± 0.13
	Liver	5.16 ± 0.19	10.08 ± 0.74	1.97 ± 0.21
LLA + CINN	Brain	2.87 ± 0.04	3.83 ± 0.52	1.34 ± 0.20
	Liver	2.89 ± 0.29	3.68 ± 0.15	1.32 ± 0.20
DLA + CINN	Brain	2.46 ± 0.29	2.79 ± 0.23	1.16 ± 0.23
	Liver	2.50 ± 0.40	2.52 ± 0.16	1.03 ± 0.10

^a^Respiratory rates in nmol O_2_/mg mitochondrial protein per min.

^b^Substrate concentrations: pyruvate (10 mM + 2.5 mM malate) and LLA or DLA (5 mM + 2.5 mM malate); CINN, α-cyano-4-hydroxycinnamate (5 mM).

State 4: The respiration state without ADP.

State 3: The respiration state with the addition of ADP.

RCR, Respiratory Control Ratio (state 3 rate:state 4 rate).

ADP/O ratio: The ratio between the amount of ADP phosphorylated and oxygen consumed.

*Significantly different from the values without substrate. Values were compared using one way ANOVA with LSD as post hoc test. α < 0.05

Note: Refer to Table 1 and 2 for the respiratory parameters without substrates or with cocktail.

## Discussion

D-lactate is present at low levels in the body and can be well utilized by the liver in both human and animal under healthy conditions [39]. However, elevated levels of blood D-lactate in several disease states [5,40] can result in D-lactate accumulation in specific tissues and potential toxicity. In fact, high D-lactate levels are associated with neurological and cardiac dysfunction [7,41]. The underlying mechanisms of such toxicities are not fully understood. In our study, we examined whether D-lactate was an efficient energy substrate for brain and heart mitochondrial and, if not, whether it could interfere with mitochondrial utilization of two major cellular energy substrates, L-lactate and pyruvate. Such interference could result in a cellular energy deficiency and, therefore, begin to explain, in part, the neurological and cardiac toxicities observed with D-lactic acidosis.

The majority of cellular ATP is generated by glycolysis and oxidative phosphorylation of pyruvate, the latter of which takes place within the mitochondria of eukaryotic cells. Impaired ATP production in mitochondria can lead to cellular energy deficiency and eventually organ dysfunctions [42]. To understand the factors that may influence mitochondrial pyruvate metabolism, isolated mitochondria are often used with measurements of respiration states, RCR and ADP/O ratios. Mitochondrial integrity and functionality was assessed with the supply of optimal substrates to assure reliable outcomes [30]. The high degree of coupling with high RCR (> 6) and a ADP/O ratio close to 3 using a typical cocktail solution (Table 1, 2, 3) demonstrated that the isolated mitochondria were functionally well preserved [43,44]. Malate was also added into our reaction mixture as it is a vital cofactor for various mitochondrial substrate transporters. It is essential for mitochondrial respiration and ensures a continuous flow of substrate across mitochondrial membranes into the matrix. Without the presence of malate, the respiration rate for state 3 is not induced by the addition of ADP with any of the selected substrates. Although mitochondria contain malic enzyme [45], malate alone did not support mitochondrial respiration in all three tissues (data not shown), which is consistent with the literature [46,47].

Although pyruvate is a key substrate for mitochondrial respiration, L-lactate can serve as a preferential substrate for mitochondrial respiration, particularly in highly metabolic tissues under conditions that have increased requirement for mitochondrial respiration [48]. In our study, ADP addition stimulated mitochondrial electron transport chain activity and oxygen consumption in all mitochondrial preparations as indicated by higher mitochondrial state 3 respiration and RCR in the presence of either L-lactate or pyruvate. Therefore, both pyruvate and L-lactate are well oxidized in rat brain, heart and liver mitochondria (Table 1, 2, 3). The heart demonstrated the highest state 3 and state 4 respiration rates with pyruvate and L-lactate as compared with brain and liver. This was expected since heart mitochondria has higher oxidative capacity compared to brain and liver [49].

RCR values can indicate the efficiency of electron transport chain coupling activity with oxidative phosphorylation [50]. Brain and heart, but not liver, mitochondria demonstrated lower RCR ratios (less than 3) with D-lactate as substrate compared to pyruvate or L-lactate (Table 2 and 3). D-Lactate also reduced oxygen consumption rates in brain and heart mitochondria but not liver and impeded efficient utilization of pyruvate and L-lactate by brain and heart mitochondria. These data coupled with findings of limited D-lactate dehydrogenase activity in brain and heart mitochondria (Figure 1) suggest that D-lactate is a poor mitochondrial respiration substrate in these tissues. Relatively high D-lactate dehydrogenase activities in liver mitochondria likely maintain efficient oxidative phosphorylation in these mitochondria in essence counteracting the effects of D-lactate noted in brain and heart mitochondria. Therefore, an accumulation of D-lactate in the brain and heart tissue may represent a secondary disorder of mitochondrial function by interfering with L-lactate and pyruvate metabolism. This has been postulated by other researchers and our study provides the first supportive experimental data for this postulate to our knowledge [28,51].

Interestingly, we also observed changes in oxygen uptake by pyruvate in rat heart, brain and liver mitochondria in the presence of L-lactate though the extents of inhibition were less compared to D-lactate (Table 1, 2, 3). Both D and L-lactate share the same mitochondrial membrane transporter and have the potential to competitively inhibit pyruvate transport into the mitochondria for energy production [28]. In the liver, both isomers of lactate can be recognized by mitochondrial lactate dehydrogenase (LDH and DDH respectively) and converted into pyruvate. Such conversion can therefore compensate for the reductions in pyruvate concentrations in the mitochondria resulting from inhibition of pyruvate transport into this organelle [52,53]. However, the degree of the compensation depends on the enzyme tissue distribution and activity. In our study, DDH activities were significantly lower in rat brain and heart mitochondria compared to liver where as LDH activities were similar between all three tissues (Figure 1). The low level of DDH in rat brain and heart may explain the strong inhibition of D-lactate on mitochondrial respiration rates using pyruvate as substrate in these two tissues.

To further investigate these findings, an LDH inhibitor (oxamate) was used to block lactate oxidation in rat brain and liver mitochondria [53]. In the presence of oxamate, mitochondrial respiration was maintained with pyruvate as substrate while mitochondrial respiration was reduced with administration of L-lactate or coadministration of L-lactate with pyruvate. These data suggest LDH plays an important role in the oxidation of L-lactate in isolated mitochondria. To investigate the possible mechanism of L- and D-lactate mediated inhibition of pyruvate metabolism, a monocarboxylate transporter (MCT) inhibitor, CINN, was used to inhibit MCT function. Marked reduction of mitochondrial respiration rates in the presence of CINN suggests a role for MCTs in the mitochondrial uptake of both isomers of lactate and pyruvate. Competitive interactions occurring at MCT may explain the inhibitory effect of D-lactate on L-lactate and pyruvate.

In conclusion, our study identified that D-lactate is a poor substrate for rat brain and heart mitochondria, but an efficient substrate for liver mitochondrial respiration. Low levels of DDH activity in rat brain and heart likely explain its poor utilization by mitochondria of these tissues. Additionally, D-lactate inhibited brain and heart mitochondrial respiration caused by pyruvate and L-lactate. L-Lactate also inhibited pyruvate induced mitochondrial respiration in liver, brain and heart but could maintain heart and brain mitochondrial respiration via LDH mediated conversion of L-lactate to pyruvate. Furthermore, an inhibitor of monocarboxylate transporters completely inhibited mitochondrial respiration in all tissues regardless of substrate. Collectively, these data suggest D-lactate inhibition of pyruvate and L-lactate mitochondrial utilization may be due, in part, to competitive inhibition of the monocarboxylate transporters responsible for the transport of pyruvate and lactate into the mitochondria. Since mitochondrial oxidative phosphorylation is the main source of ATP production in various tissues, disruption of mitochondrial respiratory function in brain and heart may compromise cellular energy status and result in toxicity. Hence, D-lactate mediated reductions in mitochondrial energy production may contribute to the neurological and cardiac toxicity associated with D-lactic acidosis. L-Lactic acidosis would not result in a cellular energy deficiency due to LDH mediated conversion of L-lactate to pyruvate by liver, brain, and heart mitochondria. Further investigation is warranted to determine the relationship between reductions in mitochondrial energy production to cellular energy deficiency and organ dysfunction.

## Competing interests

The authors declare that they have no competing interests.

## Authors' contributions

All authors contributed substantially to the body of work and have read and approved the final submitted manuscript.
